# Inferior calcar buttress reduction pattern for displaced femoral neck fractures in young adults: a preliminary report and an effective alternative

**DOI:** 10.1186/s13018-019-1109-x

**Published:** 2019-02-28

**Authors:** Wen-Feng Xiong, Shi-Min Chang, Ying-Qi Zhang, Sun-Jun Hu, Shou-Chao Du

**Affiliations:** 1 The Department of Orthopaedic Surgery, Yangpu Hospital, Tongji University School of Medicine, 450Tengyue Road, Shanghai, 200090 People’s Republic of China; 2 The Department of Orthopaedic Surgery, Tongji Hospital, Tongji University School of Medicine, Shanghai, 200090 People’s Republic of China

**Keywords:** Displaced femoral neck fractures, Inferior cortical buttress reduction, Gotfried non-anatomic reduction, Calcar support, Multiple cannulated screws

## Abstract

**Background:**

Fracture reduction quality is of paramount importance for osteosynthesis. The aim of this study was to report the outcome of an inferior cortical buttress non-anatomic reduction pattern and internal fixation for displaced femoral neck fractures (Garden types III and IV) in young adults.

**Methods:**

A retrospective analysis of 46 displaced femoral neck fractures was performed, which were treated by closed reduction and internal fixation with parallel cannulated screws. There were 20 males and 26 females, with an average age of 50.3 years (19–60). According to the inferior cortical reduction quality seen in recorded intraoperative fluoroscopy, the patients were divided into two groups. Group I (*n* = 30) was anatomic cortical apposition as the two inferior cortices were smoothly contacted, and group II (*n* = 16) was buttress cortical apposition as the two inferior cortices were located in positive support contact (Gotfried reduction pattern). With a mean follow-up of 22.0 months, femoral neck length, neck–shaft angle, and clinical outcomes were compared.

**Results:**

Thirty-nine patients (84.8%) achieved uneventful fracture union. Complications occurred in seven patients, six in group I (20%) and one in group II (6.3%), including displacement to varus, neck shortening, early fixation failure, nonunion, and avascular necrosis of the femoral head. No significant difference existed in the complication rate between the two groups (*p* = 0.216). Four patients (13.3%) in group I were converted to prosthetic replacement, but none in group II.

**Conclusions:**

For closed reduction and fixation of displaced femoral neck fractures in young adults, an inferior cortical buttress reduction pattern, though non-anatomic, can produce sustainable fracture stability and predictable clinical outcomes.

## Background

Femoral neck fractures in young adults usually result from high-energy trauma [1]. These injuries may involve a displaced fracture pattern, resulting in instability of the fracture site, and this may be associated with complications including fixation failure, neck shortening, nonunion, or head avascular necrosis of the femoral head [2, 3].

**Fig. 1:**
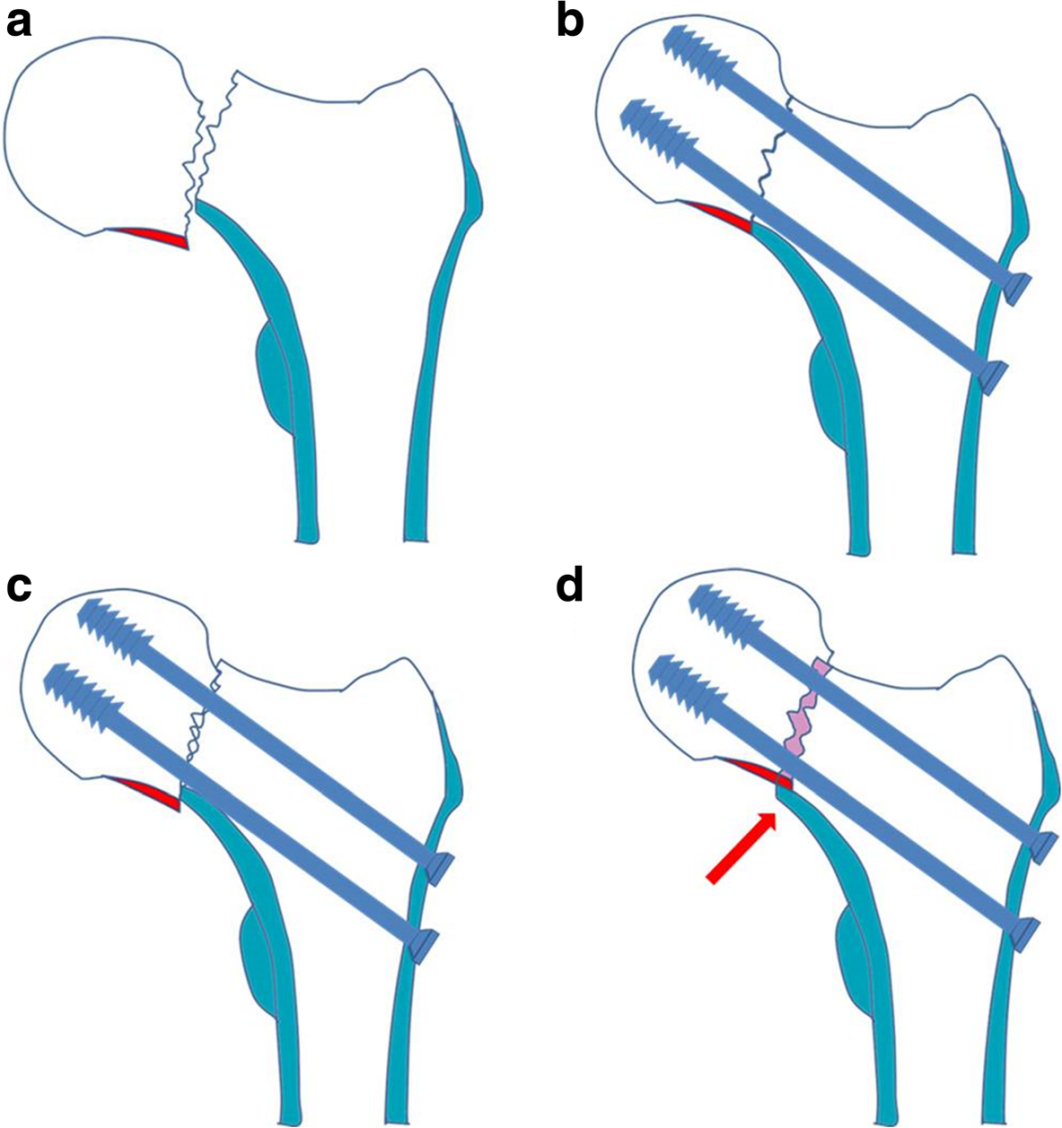
Schematic drawing to show the inferior cortical apposition pattern after manual reduction in displaced femoral neck fractures. **a** The displaced femoral neck fracture. **b** Anatomic cortical apposition with smooth inferior cortex apposition. **c** Negative cortical apposition lost calcar support. **d** Positive cortical apposition obtained calcar support. The proximal head-neck fragment is slightly superiorly displaced intentionally (less than one cortex thickness or 4 mm) to the distal fragment (red arrow), so that the inferior cortex of the proximal head-neck fragment (red color) can be buttressed by the calcar cortex of the distal neck fragment (blue color). After screw fixation, the femoral head-neck is dual supported by the fixation screw and the calcar cortex, and a sustainable stability is restored

Besides the degree of initial fracture injury, anatomic reduction is thought to be the key for successful uneventful fracture union. There is no doubt that excellent reduction quality is the most important procedure for the treatment of displaced femoral neck fracture, before any kind of internal fixation is carried out. However, a true exact anatomic reduction may be difficult to justify in closed reduction and fluoroscopy monitoring. In 2012, Gotfried et al. [4] proposed a non-anatomic cortical buttress reduction pattern with inferior calcar support (Fig. 1) and obtained favorable clinical results in a small number of patients [5]. However, no other report till now was presented in the literature.

We retrospectively reviewed our patient data and attempted to evaluate the value of this new cortical buttress reduction pattern and compare the results with the anatomic reduction.

## Materials and methods

### Patients

After Institutional Review Board approval (No. 2017ZRKX-013), we retrospectively reviewed our data from January 2014 to January 2017. The inclusion criteria were as follows: (1) 60 years of age or less, (2) isolated and displaced fractures (Garden types III and IV), (3) closed reduction and fixation with multiple cannulated screws (6.5 mm in diameter) in inverted triangle or diamond configuration, and (4) more than 1 year of follow-up. Patients with a pathological fracture and a malignant disease, open fracture, previous fracture, symptomatic osteoarthritis of the hip before injury, and rheumatoid arthritis, as well as those without complete sets of preoperative, postoperative, and follow-up radiographs, were excluded.

There were 46 patients who met the criteria and were included in this study. The subject group was comprised of 20 males and 26 females, with an average age of 50.3 years (range, 19–60 years). Personal age at the time of surgery, body mass index (BMI), score of  American Society of Anesthesiologists (ASA), and follow-up period were recorded retrospectively after patient inclusion. All information was recorded, and all evaluations were performed by the senior visiting orthopedic staff.

### Fracture management

**Fig. 2:**
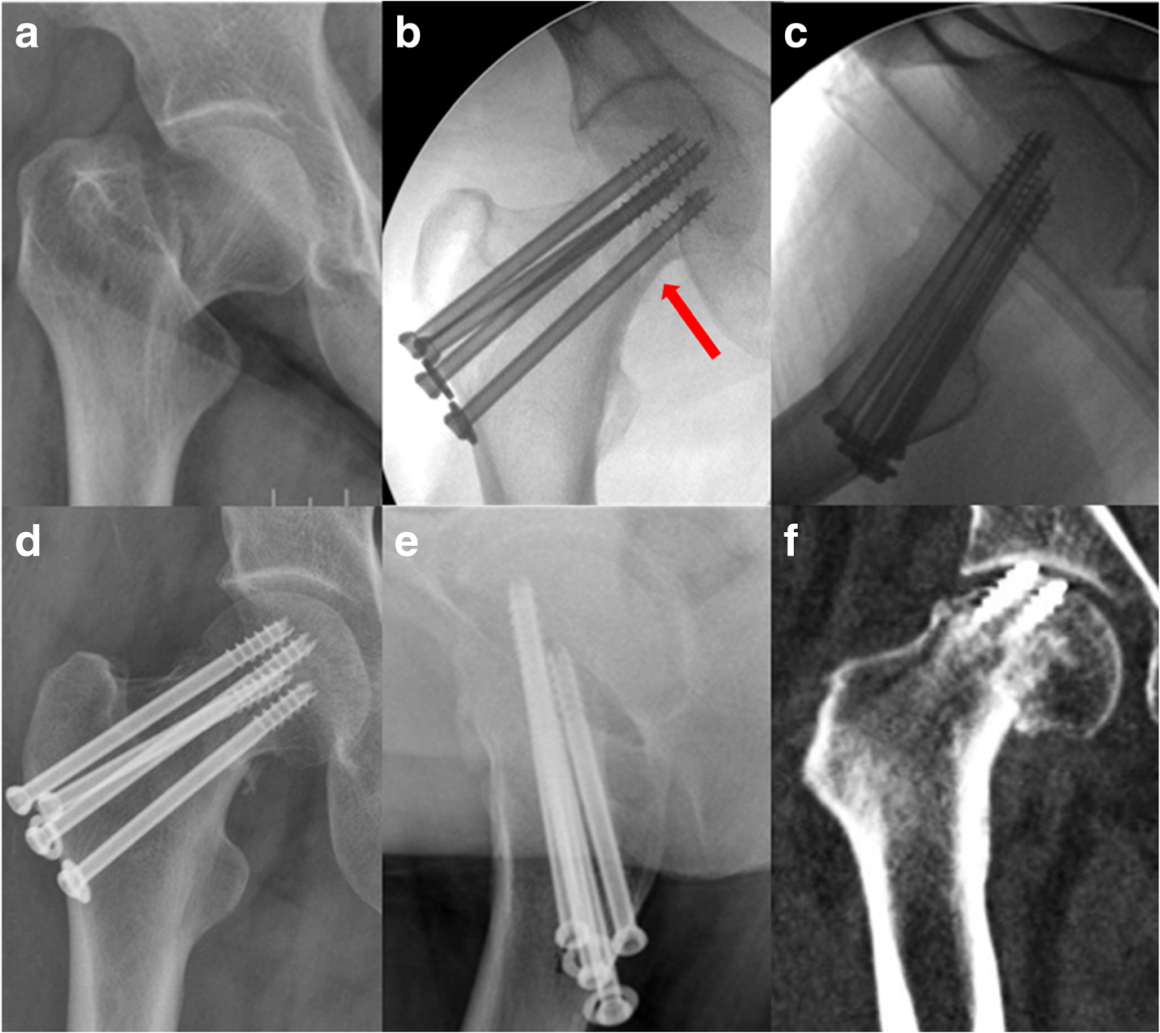
AP view of a displaced femoral neck fracture in a 55-year-old female (**a**). The fracture obtained smooth anatomic reduction (red arrow) according to intraoperative fluoroscopies (**b**, **c**), and the fracture changed into slightly varus (≤ 10°) 3 days postoperatively (**d**, **e**). CT showed the fracture displaced to varus (> 10°) 4 months postoperatively, which resulted in early failure as the screws cut out from the femoral head (**f**)

**Fig. 3:**
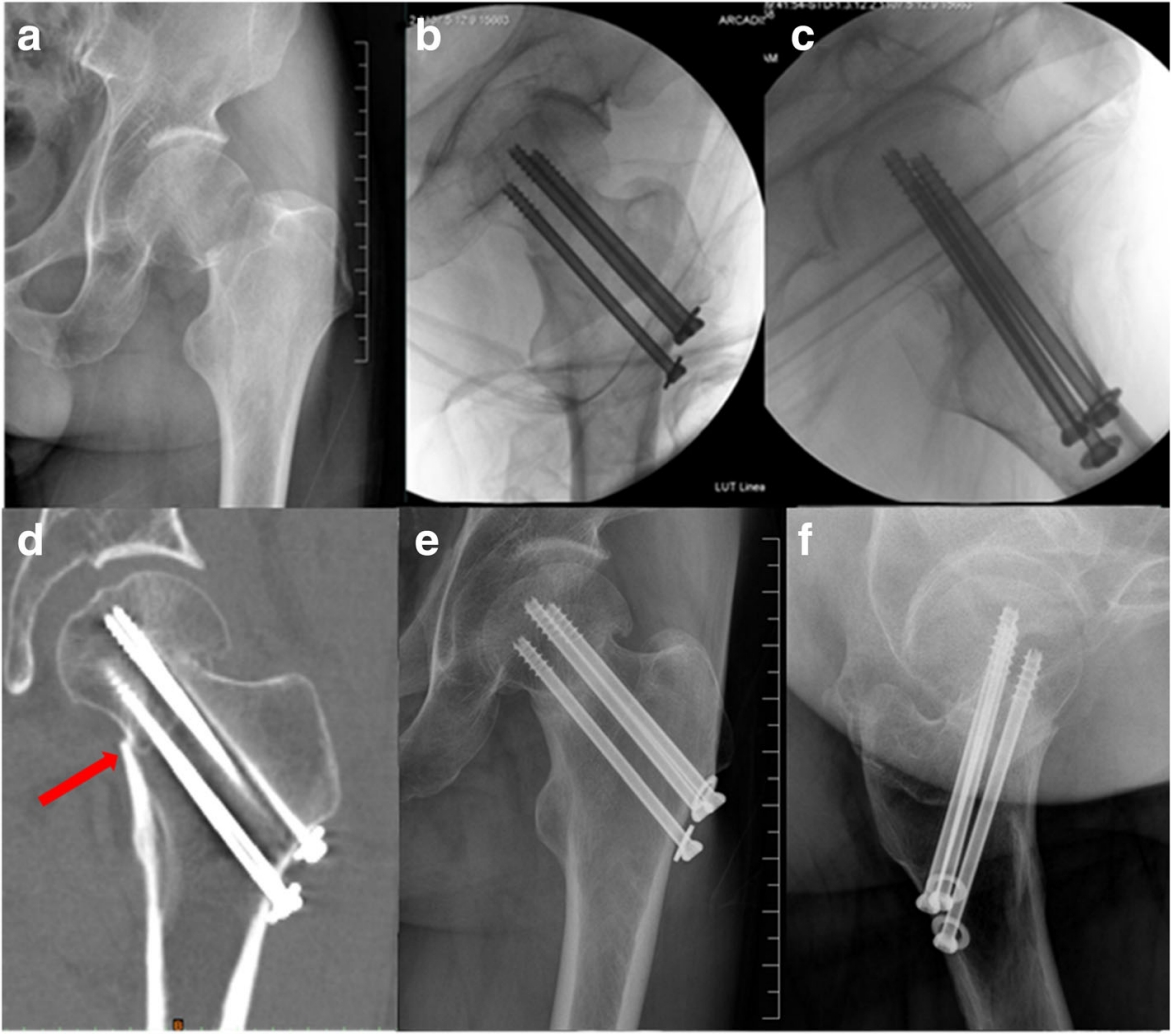
(**a**) A 56-year-old male suffered from displaced femoral neck fracture. (**b**, **c**) Intraoperative fluoroscopies showed that inferior cortical buttress pattern reduction was obtained. (**d**) CT scanning demonstrated the inferior medial cortices were resisted on a positive position (red arrow). (**e**, **f**) In six months follow-up, the fracture healed

**Fig. 4:**
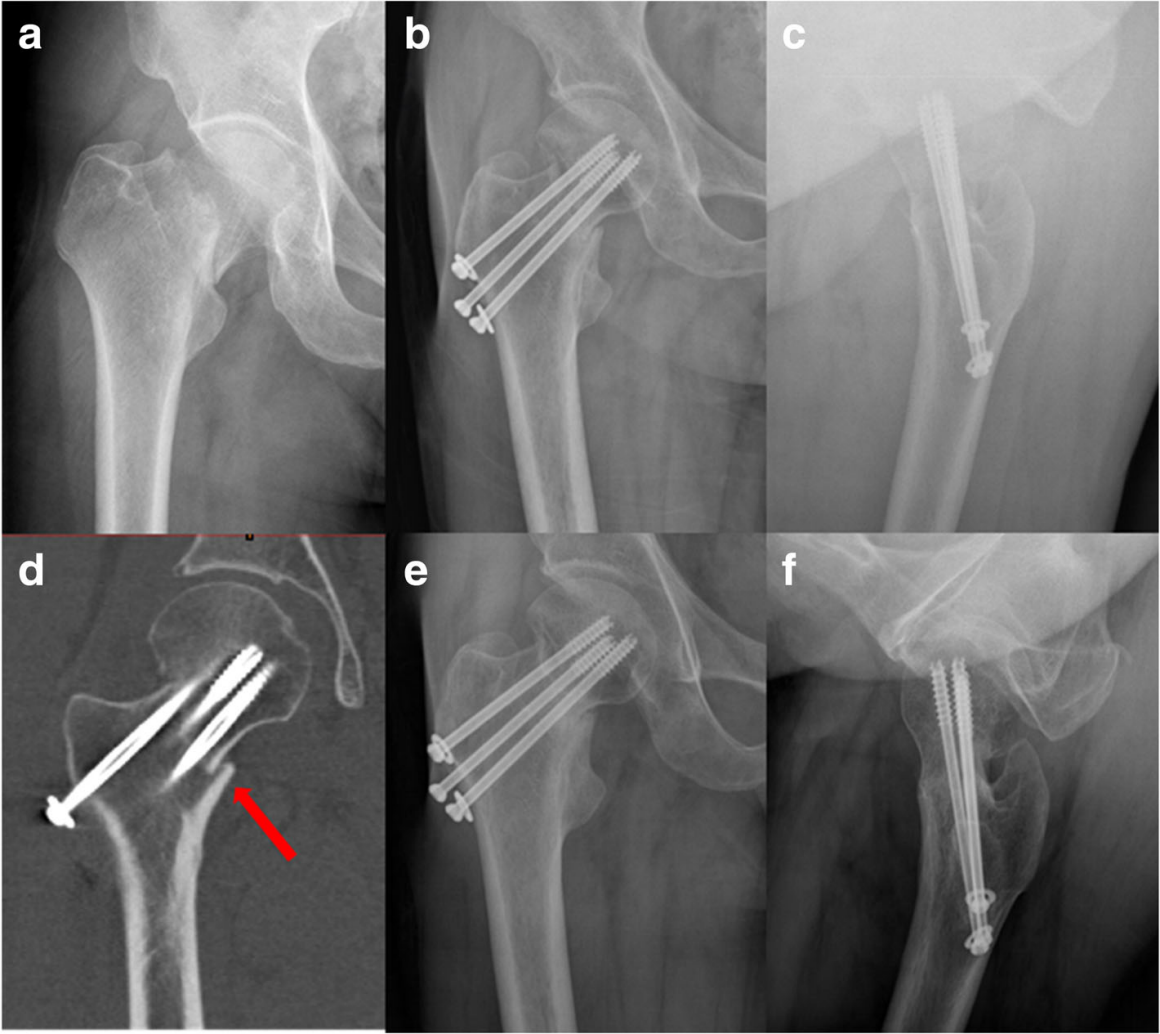
AP view of femoral neck fracture in a 53-year-old male (**a**). The fracture was reduced with an inferior cortex support pattern (**b**, **c**). CT showed cortex buttress (red arrow, **d**). Although gradually displaced to varus (> 10°) and shortening of the femoral neck, the fracture eventfully united and the patient restored full-weight-bearing walk in 6 months postoperatively (**e**, **f**)

The patients were positioned on a fracture table, and fluoroscopy with an image intensifier was used routinely to guarantee the quality of the procedure. As a general rule in practice, firstly, we performed closed manual reduction and evaluated the fracture reduction quality by fluoroscopy. Besides Garden alignment (residual angle) in AP and lateral views, we accepted two cortical apposition patterns: anatomic inferior smooth reduction (Fig. 2) and non-anatomic inferior buttress reduction (Figs. 3 and 4). If closed reduction was not satisfied after two attempts, open reduction through Watson-Jones approach was carried out and those patients were excluded from this study.

Postoperatively, sitting was encouraged at the first day, non-weight-bearing ambulation with the aid of a walker frame was allowed after 1 or 2 weeks as long as the pain was tolerated. Patients were encouraged to partial weight bearing 6 weeks after the operation. For patients in whom radiographic and clinical healing appeared to be progressing towards union at 6 weeks, weight bearing was advanced slowly from toe touch to partial weight bearing with a walker frame over the subsequent 6 weeks. When the clinical and radiological signs of union were presented, full weight bearing was allowed.

The patients were routinely followed up at 6 weeks, 3 months, 6 months, and 12 months postoperatively and annually thereafter, and follow-up plain radiographs were obtained at every follow-up visit. In addition, as postoperative lateral radiography often did not meet the standard criteria, some patients were further evaluated with CT scanning.

### Radiographic assessment

The quality of fracture reduction was evaluated using the recorded images of intraoperative fluoroscopy. All enrolled patients were divided into two groups: those reduced with anatomic reduction (group I) and those with non-anatomic cortical buttress reduction (group II).

Garden alignment index angles on intraoperative fluoroscopy (both AP and lateral views) were measured to elevate the degree of residual angulation. Two parameters were calculated to detect possible changes on intraoperative fluoroscopy and postoperative follow-up radiographs. The femoral neck–shaft angle (FNSA), which is the angle between the two axes of head-neck and shaft medullary, was measured. Changes of more than 10° were defined as displacement to varus. Femoral neck length (FNL) was measured according to the method introduced by Zlowodzki et al. [6], and more than 5-mm changes were defined as shortening.

Early failure was defined as loss of reduction requiring a reoperation within 6 months. Nonunion was defined as a clearly visible fracture line at 12 months postoperatively.

### Statistical analysis

All statistical calculations were performed using SPSS version 19.0 (SPSS Inc. Chicago, IL, USA). Basic descriptive statistical analyses were used to describe the patient population and treatment outcomes. The Mann-Whitney *U* test was used for continuous data. The *χ*^2^ test or Fisher exact test was used for categorical variables. Statistical significance was defined as *p <* 0.05.

## Results

For the group as a whole, the mean follow-up time was 22.0 months (range, 12–36 months). No statistical difference in gender, age, body mass index (BMI), American Society of Anesthesiologists (ASA) score, and follow-up time was observed between the two groups (Table 1).

**Table 1:** Demographic data of patients

Item	Total (*n* = 46)	Group 1 (*n* = 30)	Group 2 (*n* = 16)	*p*
Male/female	20/26	13/17	7/9	0.610
Age, years	50.3 ± 9.6	49.2 ± 11.0	52.4 ± 5.9	0.594
BMI	22.9 ± 1.6	22.6 ± 1.7	23.5 ± 1.2	0.074
ASA score	1.3 ± 0.5	1.3 ± 0.5	1.4 ± 0.5	0.609
FU time, months	22.0 ± 10.4	22.9 ± 11.7	20.1 ± 7.2	0.871

Note: Group 1 = anatomic reduction group. Group 2 = Gotfried reduction group. Data are expressed as mean ± SD

*BMI* body mass index, *ASA* American Society of Anesthesiologists, *FU* follow-up

There were 30 cases classified as cortical anatomical reduction, and 16 cases as cortical buttress reduction. The Garden alignment index angles were 165.1° in average in AP view and 178.2° in lateral view. No statistical difference was revealed between the two groups (*p* = 0.104) by the Mann-Whitney *U* test. During follow-up, 39 patients (84.8%) achieved fracture union uneventfully. Complications occurred in seven patients, six in group I (20%) and one in group II (6.3%). No significant difference existed in the complication rate between the two groups (*p* = 0.216). Four patients (13.3%, all in group I) were converted to prosthetic replacement. However, the Fisher exact test revealed no significant difference between the two groups (*p* = 0.168) (Table 2).

**Table 2:** Comparison of complications of the two groups

Item	Group 1 (*n* = 30)	Group 2 (*n* = 16)	*p*
Complications	6 (20%)	1 (6.3%)	0.216
Displacement to varus (> 10°)	3 (10%)	1 (6.3%)	/
Shortening (> 5 mm)	3 (10%)	1 (6.3%)	/
Nonunion	2 (6.7%)	0	/
Early failure	1 (3.3%)	0	/
AVN	1 (3.3%)	0	/
Conversion to prosthetic replacement	4 (13.3%)	0	0.168

The mean varus change of the femoral neck–shaft angle was 3.4° (Table 3). Considering varus displacement in postoperative follow-up, no significant difference was noted between the two groups (*p* = 0.715). Using 10° as a cutoff criterion, three fractures (10%) displaced to varus in group I. In a female patient, the anatomic reduction changed into slightly varus (≤ 10°) in 3 days and gradually had a screw cutout in 4 months (Fig. 2). The patient accepted conversion to prosthesis. The remaining two gradually displaced to varus, both of them were diagnosed with fracture nonunion 6 months postoperatively and accepted conversion to total hip arthroplasty (THA). In group II, one patient gradually displaced to varus and had femoral neck shortening, but the fracture was eventually united without discomfort in 6 months (Fig. 4).

**Table 3:** Comparison of radiological assessment in the two groups

Parameters	Total (*n* = 46)	Group 1 (*n* = 30)	Group 2 (*n* = 16)	*p*
Garden alignment AP angle	165.1 ± 3.3	164.5 ± 3.6	166.2 ± 2.5	0.104
Garden alignment lateral angle	178.2 ± 1.5	178.2 ± 1.5	178.0 ± 1.5	0.714
Varus change of the FNSA, degrees	3.4 ± 3.9	3.5 ± 4.2	3.1 ± 3.4	0.715
Shortening length of the femoral neck, mm	3.0 ± 2.8	3.3 ± 3.4	2.6 ± 1.3	0.914

Note: Garden alignment index angles (AP and lateral views) were measured on intraoperative fluoroscopy

*FNSA* femoral neck–shaft angle

The mean shortening length of the femoral neck was 2.7 mm (Table 3). Using 5 mm as a criterion, three patients in group I had femoral neck shortening. One was diagnosed with avascular necrosis (AVN) 3 years postoperatively and accepted conversion to THA. The other two did not complain any discomfort with neck shortening.

## Discussion

In the treatment of young adult femoral neck fractures, fracture initial displacement and the quality of reduction are the major determinant factors for outcomes. Anatomic reduction is the key to success in fracture union. For grading the quality of femoral neck fracture reduction, two parameters are used: the degree of residual angulation (Garden alignment) and the amount of displacement (cortex apposition, regardless of direction). Excellent is defined as < 2 mm of displacement and < 5° of angulation in any plane, good as 2 to 5 mm of displacement and/or 5° to 10° of angulation, fair as > 5 to 10 mm of displacement and/or > 10° to 20° of angulation, and poor as > 10 mm of displacement and/or > 20° of angulation [7]. Based on the above criteria, Haidukewych et al. [7] reported that even with good to excellent fracture reduction in 46 patients, 11 (24%) were associated with the development of osteonecrosis and 2 (4%) were associated with the development of nonunion. Gardner et al. [8] noted that the rate of complication was 10% with “excellent” reduction quality, and 26% with “good” reduction quality. Liporace et al. [9] reported that 8 of the 59 (14%) vertical femoral neck fractures with good to excellent reduction quality had fracture nonunion.

However, the so called *anatomic reduction* in intraoperative fluoroscopic monitoring does not guarantee good outcomes postoperatively. During healing of femoral neck fracture, both bone resorption and shear forces of the fracture site can result in secondary sliding and displacement, which tend to cause femoral neck shortening and neck–shaft angle varus, even though the fracture was reduced in anatomic reduction quality [5]. It is well known that the varus displacement is a paramount predictor for failure in femoral neck fractures [10], and femoral neck shortening will decrease the purchase of screws and can also lead to failure [3]. Bone resorption is inevitable during healing of femoral neck fracture, and shear forces can hardly be controlled except for limited weight bearing. Therefore, techniques avoiding the negative affect of bone resorption and shear forces are reasonable to reduce the incidence of complications.

We think the so called *anatomic cortical reduction* shown in intraoperative fluoroscopy may actually contain three sub-conditions: some are exact anatomic cortical reduction, others slightly negative cortical reduction, and still others slightly positive reduction. But as the image resolution of intraoperative fluoroscopy was limited, those three sub-conditions were hard to be distinguished from one another. Negative cortical apposition means no cortical support and the head-neck fragment has a clear tendency to displace in a varus position [11]. After bone resorption of the fracture site, a slightly varus apposition may become truly varus and initiate displacement to cause fixation failure (Fig. 2) or fracture nonunion. This may explain the complications in our group I. We suggest that even though anatomic reduction is seen in intraoperative fluoroscopies, immediate postoperative radiographs and close postoperative follow-up are required to detect possible reduction changes. Intraoperative CT scanning is accurate to detect reduction quality, but it is not applicable in most hospitals.

Gotfried introduced the cortical buttress reduction, which was a non-anatomic functional reduction pattern [4]. The proximal head-neck fragment is slightly displaced superiorly to the distal fragment intentionally (less than one cortex thickness or 4 mm), so that the head-neck is supported and is upheld by the inferior calcar cortex. And simultaneously, the head-neck fragment is usually located in a slight valgus position. After screw fixation, the femoral head-neck is dual buttressed by the fixation screw and the calcar cortex, and a sustainable fracture stability is restored.

In a Gotfried reduction pattern, the head-neck fragment was further supported by the inferior femoral neck cortical calcar, unlike in an anatomic reduction pattern, in which the head-neck fragment was only supported by the fixation screw. It can resist the vertical shear forces by inferior cortical contact, and transfer the shear forces to compress the bone substance of the fracture site, providing both mechanical and biological environment for the fracture stability and healing. Even though the fracture displaced to varus because of bone resorption, fracture union can be anticipated because of the compression of the fracture site (Fig. 4), i.e., an inferior cortical buttress reduction pattern can reduce the negative affect of fracture site resorption. In our group II of 16 patients, fracture union was eventfully achieved in all cases, with only one patient who developed hip varus.

In 2015, Mir and Collinge hypothesized the application of a small medial buttress plate to augment fixation in vertical shear femoral neck fractures [12]. In 2017, Ye et al. [13] reported the preliminary clinical results in 28 cases of vertical femoral neck fractures treated with open reduction and three cannulated screws and augmented with a medial buttress plate. Despite all fracture reduction achieved Garden’s alignment index grade 1, reduction loss with backing out of the cannulated screws was found in three cases, with plate screws broken in one case in 3 months of follow-up. The medial buttress plate fixation can effectively resist shearing force of the fracture site to avoid displacement to varus; however, it cannot prevent the negative affect of bone resorption and needs additional exposure.

There are several advantages of an inferior cortical buttress reduction pattern. It emphasizes the direction of cortical apposition, using the inferior calcar cortex to uphold the head-neck fragment. It is a non-anatomic pattern and is easy to achieve by closed maneuver, and no need of additional operation. It has favorable biomechanical properties after fracture site resorption and sliding. Our preliminary results show that it is an effective clinical alternative to the anatomic reduction.

Some limitations of the present retrospective study must be acknowledged. Firstly, it is a preliminary report with a small number of cases. Secondly, we found no case of AVN in group II. It may be due to the short follow-up period. Larger-scale and long-term follow-up studies are needed to confirm the ultimate outcome with a Gotfried non-anatomic cortical buttress reduction pattern.

## Conclusion

Our preliminary study showed that fracture reduction with an inferior cortical buttress pattern, though non-anatomic, can produce excellent outcomes in closed fixation of displaced femoral neck fractures. Both calcar support reduction and anatomic reduction are acceptable in clinical practice.

## Abbreviations

ASA: American Society of Anesthesiologists
BMI: Body mass index
FNL: Femoral neck length
FNSA: Femoral neck–shaft angle
FU: Follow-up
THA: Total hip arthroplasty
